# International Dental Federation

**Published:** 1903-11-15

**Authors:** Ch. Godon, E. Sauvez

**Affiliations:** President. Office of the Secretary-General, 45 Rue De La Tour D'Auvergne, Paris; Secretary-General. Office of the Secretary-General, 45 Rue De La Tour D'Auvergne, Paris


### INTERNATIONAL DENTAL FEDERATION.

(Circular to the Presidents of National Dental Societies.)

Office of the Secretary-General,
45 Rue de la Tour d'Auvergne,
Paris, September 18, 1903.

*Dear Sir and Honored Confrere* The International Dental Federation at its meeting held in Stockholm in August, 1902, in accordance with the powers that had been conferred upon it, decided that the Fourth International Dental Congress be held at St. Louis, Missouri, in August, 1904, at the time of the holding of the St. Louis Universal Exposition. The decision thus reached followed the receipt of invitations regularly addressed to the F. D. I. by the National Dental Association, the Odontological Society of St. Joseph, the National Association of Dental Examiners' the Odontographic Society of Missouri and Western Kansas, the Society of Dental Science of St. Louis, the Committee appointed by the Missouri State Dental Association, the Dental Society of St. Louis, the City of St. Louis, the Government of the State of Missouri, and the authorities of the Louisiana Purchase Exposition. This decision was confirmed at the session of the F. D. I. held in Madrid in April, 1903.

The officers of the Executive Council of the F. D. I., in accord with the authorities of the Exposition and the Committee of Organization of the Congress, have determined the conditions under which the Federation will take part in the organization of said Congress, and we are now officially advised that the Fourth International Dental Congress will be held in the city of St. Louis in 1904, from August 29th to September 3d, inclusive.

The purpose of this circular is to inform you that the International Dental Federation has decided to lend its entire support to the organizers of the Fourth International Dental Congress, and in view of assuring the perfect success of the Congress, we therefore request you to appeal to the several dental societies in your country to take part in the said Fourth International Dental Congress.

It seems unnecessary to call your attention to the importance of all dental societies the world over being appropriately represented at this gathering, both scientifically and professionally. We think it, however, desirable to call attention to matters which the delegates of the different federations and national societies will be called upon to discuss with reference to the organization of the second term of the International Dental Federation —that which will be comprised, namely, in the period between the Fourth and the Fifth International Dental Congresses.

During the first working period of the F. D. I., comprised between the Third International Dental Congress held in Paris in 1900 and that of St. Louis to be held in 1904, the members of the Executive Council of the F. D. I. who were appointed in Paris by your representatives have fulfilled to the best of their abilities the mission with which they had been entrusted by the members of the Third International Dental Congress, of which you may have been able to judge from perusal of the printed transaction of its different meetings.

They have assured the holding of a Fourth International Dental Congress (resolution No. 13).

They have created an International Commission of Education, which presented a program of international dental education at the sessions held in London, Cambridge, Stockholm, and Madrid (resolution No. 16).

The International Commission of Dental Hygiene, organized at the Cambridge session, will, at the St. Louis meeting, complete the program of international dental hygiene to be recommended to the public authorities.

Other projects of interest to the evolution of dentistry—such, for instance, as the publication of an international dental review—are being carefully studied in view of future realization. Reports have been prepared and presented on the federation of schools, and other propositions are now under consideration, such as the creation of a universal nomenclature, and also of a code of ethics to be universally accepted. The execution of these projects will fall on our successors in the subsequent terms of the F. D. I.

It will be the duty of the delegates to the St. Louis Congress to ratify the constitution of the F. D. I., to introduce such amendments as may be necessary, to appoint the members that will represent the different countries in the Executive Council, and to decide on the program of the second period of the F. D. I.

Appreciating the importance of the great international gathering to be held in St. Louis in August, 1904, we are convinced that you will be able to induce the National Dental

Association and the other dental societies of your country to take part in the Congress by sending delegates and by contributing scientific papers.

We request that this circular be published in all the dental journals of your country.

Please accept, dear sir and *confrere*, the assurances of our fraternal sentiments.

DR. CH. GODON, *President.*

DR. E. SAUVEZ, *Secretary-General.*

